# Pitfalls at Chemistry of Adenoviral Vector Vaccine against COVID-19 and How to Circumvent it

**DOI:** 10.34172/apb.2022.024

**Published:** 2021-08-07

**Authors:** Amr Ahmed, Mohammad Nezami, Abdullah Alkattan

**Affiliations:** ^1^Department of Public Health, Tuberculosis Program, First Health Cluster, Ministry of Health, Riyadh, Saudi Arabia.; ^2^President and CEO of Sahel Oncology LLC, Orange Coast Medical Center of Hope Inc. 496 Old Newport Blvd. #7 Newport Beach, CA 92612, USA.; ^3^Biomedical Sciences department, Faculty of Veterinary Medicine, King Faisal University, Saudi Arabia.

## Dear Editor,

The coronavirus disease 2019 (COVID-19) is a pandemic outbreak that causing a number of deaths reached over 3 million since December 2019. Despite the approval and production of several vaccines against SARS-CoV-2 infection, many countries are still suffering from daily deaths due to COVID-19. Although the vaccination’s success in reducing the cases infected with SARS-CoV-2, many people globally are still not immunized against COVID-19 due to their doubts about the risks and benefits of the vaccines. Here, we would like to spotlight on vaccines’ excipients and how to evade the hurdles of some COVID-19 vaccines.

ChAdOx1 nCoV-19 vaccine is an adenovirus vector vaccine designed to provoke immunity against SARS-CoV-2. This vaccine contains several inactive ingredients, including sodium chloride, magnesium chloride hexahydrate, ethanol, sucrose, and ethylene diamine tetra acetic acid (EDTA).^1^

EDTA is a very efficient zinc chelator which is utilized commonly in protein interaction research. Exposure to EDTA even in lower concentrations may cause extreme stripping of zinc from many proteins, including zinc-binding proteins that are described as a component of the largest and most complex gene superfamily in metazoans and the most prevalent category of transcription parameters. The zinc dissociation rates can vary greatly among these proteins. Furthermore, zinc-binding domains may not be refolding into their true native structure after EDTA removal, The addition of extra zinc did not restore C/H1 to its original structure, and the binding between C/H1 and HIF1alpha is abolished.^2-4^ Research by Matt et al indicated that EDTA treatment of native cysteine/histidine rich region 1 (C/H1) causes aggregation and irreversible denaturation, and that particular concern their finding that unfolded C/H1 results in non-specific protein-protein interactions.^4^

Nevertheless, the EDTA-induced thrombocytopenia is a risk phenomenon caused by EDTA-dependent anti-platelet auto-antibodies that identify antigens modified by EDTA. Few case reports indicating that thrombocytopenia and thrombosis events after receiving the vaccines containing EDTA cannot be ruled out.^5,6^ The cation chelation by EDTA causes changes in the shape of the platelet membrane glycoprotein IIb-IIIa complex and showed an obscure epitope that becomes available for autoantibodies.^7^ This leads to platelet clumping and aggregation in vitro, leading to low platelet counts, which slightly increase in white blood cells.^8^

Another issue is the adenovirus in the vaccine. It can induce thrombocytopenia, a potentially severe complication of gene therapy protocols by this kind of vector. While studying the interaction between adenovirus and platelets, we proved adenovirus platelet attachment by detecting Coxsackie adenovirus receptor. We concluded that the interaction between platelets and adenovirus causes platelet activation and quick appearance of P-selectin on the platelet’s surface; this triggers the formation of platelet-leukocyte aggregates. This interaction is vital since it promotes leukocyte rolling on the endothelium and slows platelets that excrete the ligand in an inflammatory setting. The thrombocytopenia appearance in 24 hours of adenovirus administration reveals that this is a response to the virus. Studies of the administered adenovirus show that liver macrophages clear most of the virus within 24 hours, the same amount of time that platelets take to be cleared. According to this observation, we can conclude that the adenovirus and the activated platelets may be cleared with the same mechanism. For further evidence, we noticed the adenovirus particles’ appearance in the liver macrophage 10 minutes after the injection.^9^

Many interactions are acknowledged to occur between Ad capsid and non-cellular and cellular blood components, in addition to the complement system. For instance, Ad vectors interact with platelets, causing thrombocytopenia.^10,11^

The most difficult obstacle for Ad vectors comes from the stimulation of an intense immune response, possibly caused by reacting with antigen-presenting cells like dendritic cells and macrophages; this causes the release of pro-inflammatory cytokines/chemokines like tumor necrosis factor-α, interferon γ, inducible protein-10, RANTES, and interleukin 6 (IL-6).^12^

Because the spleen is the primary location of cytokine and chemokine synthesis, such as interleukin-6, decreased absorption of PEGylated vectors by the spleen could indicate why lower IL-6 concentrations were seen following PEGylated Ad vector delivery. PEGylated Ad vectors demonstrated much-decreased spleen (3-fold lower) and kidney transduction, according to De Geest et a1^13^ (2.5-fold lower). PEGylation further prevented thrombocytopenia from developing. Finally, combining PEGylation of adenoviral vectors with methylprednisolone injection increases the therapeutic index of adenoviral gene transfer.^13^ PEGylation has already resulted in a 70% reduction in serum IL-6 concentrations.^14^

We noted that the study by Ewer et al studied immune response by a single dose of ChAdOx1 nCov-19, not included the test of Il-6.^15^

We conclude from previous studies that the PEGylating of adenoviral vectors can be promise technology as safety profile as significantly reduced IL-6 and liver toxicity and how avoiding the pitfalls of chemistry and virology, so the PEGylation since the first time in 1999 introduced by O’Riordan et al^16^ need more advancements.

## Ethical Issues

Not applicable.

## Conflict of Interest

None.
